# Whole mitochondrial genome sequencing and phylogenetic analysis of Gangetic mystus (*Mystus cavasius*)

**DOI:** 10.1080/23802359.2024.2427106

**Published:** 2024-11-15

**Authors:** Suvra Roy, Pranaya Kumar Parida, Ramya Kumar, Vikash Kumar, Dibakar Bhakta, Bijay Kumar Behera, Basanta Kumar Das

**Affiliations:** a Aquatic Environmental Biotechnology (AEB) Division, ICAR-CIFRI, Barrackpore, India; b ICAR-Central Inland Fisheries Research Institute (CIFRI), Barrackpore, India; c Regional Centre of ICAR-CIFRI, Bangalore, India

**Keywords:** Mitochondrial genome, *Mystus cavasius*, indigenous catfish, phylogeny, next-generation sequencing

## Abstract

*Mystus cavasius*, known as Gangetic mystus, is a freshwater indigenous catfish. The present study represents the first-ever complete mitochondrial genome sequencing of *M. cavasius*. The whole mitochondrial genome size is 16,554 bp (GenBank accession number OR018997). The mitogenome organization consists of total 37 genes, with 13 protein-coding genes, two rRNA, 22 tRNAs, and a D-loop regulatory region. Among these, 28 genes are coded on the heavy strand, while eight tRNA genes and ND6 are encoded separately. Phylogenetic analysis reveals that *M. cavasius* clusters with other *Mystus* species and bagrid catfishes. This work contributes valuable insights into structural characterization and phylogenetic relationships.

## Introduction

Gangetic mystus (*Mystus cavasius*) is a freshwater catfish that belongs to the family Bagridae in Siluriformes order. *M. cavasius*, locally known as ‘gulshe tengra’, is an important small indigenous fish (SIF) species in India that holds several social and traditional importance. It has been reported to be dispersed along the Indian subcontinent including Bangladesh, Pakistan, Nepal, Sri Lanka, Thailand, and Myanmar (Talwar and Jhingran 1991; Chakrabarty and Ng 2005). It is a eury-omnivorous and hardy fish, tolerating many environmental factors. The breeding season of this fish extends from April to September, with peak spawning activity in July (Roy et al. 2022). This fish species has become popular due to its finest taste and nutritional profile (Ashashree et al. 2013; Hossen et al. 2014). *M. cavasius* has been considered a potential aquaculture species because of its characteristic high stocking density culture, short life cycle, faster growth, and high market demand (Rahman et al. 2013; Hossain et al. 2016). Recently, it has been documented that *M. cavasius* is a candidate indigenous ornamental fish species for export in India (Gupta 2014). However, the Bagrid catfish evolutionary relationships are poorly understood because of the limited genomic data employed and the ongoing re-description and reporting of new species (Darshan et al. 2019). Molecular studies have not yet been used extensively as systematic tools to study phylogeny and species differentiation. Since complete genome sequences offer essential insights into many deep-level phylogenetic questions, the mitochondrial genome has evolved into a potent molecular marker for species classification and phylogenetic analyses (Prabhu et al. 2020). Hence, the present study attempts to characterize the whole mitochondrial genome of *M. cavasius* for the first-time using Illumina next-generation sequencing technology.

## Materials and methods

### Sample collection and DNA extraction

**Figure 1.**
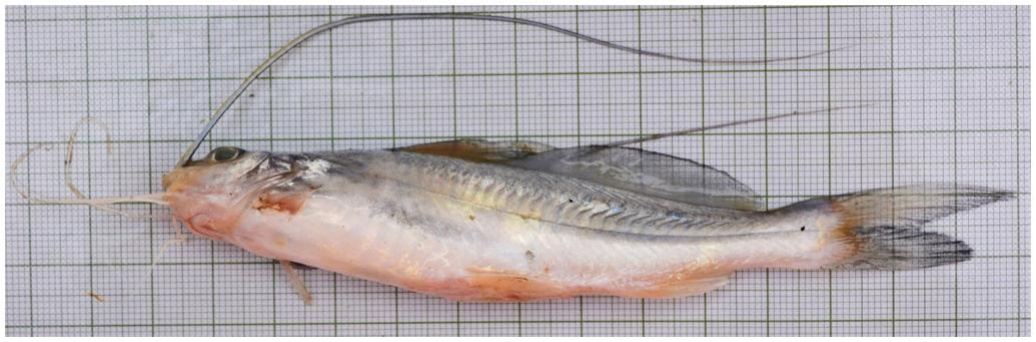
*Mystus cavasius* reference fish specimen (135 mm length) collected from the Balagarh region (23.122444°N, 88.463278°E) of the lower part of the Ganga River, West Bengal, India. Four pairs of barbels and maxillary pair crossed the base of the caudal fin. Dorsal portion grey, underneath yellowish. A black spot after the operculum. Adipose, caudal, and dorsal fins have melanophores (the authors Dibakar Bhakta and Suvra Roy have obtained the fish specimen and took the photo of species reference image).

*M. cavasius* (Hamilton, 1822) specimen used in the present work was collected from the Balagarh region, Ganga River (23.122444°N, 88.463278°E), West Bengal, India, in 2022. The fish was identified using documented morphometric and meristic characteristics (Talwar and Jhingran 1991) (Figure 1). For future reference, a specimen was stored at ICAR-Central Inland Fisheries Research Institute laboratory (http://www.cifri.res.in/; AK Jana, asim.jana121@gmail.com and Director, ICAR-CIFRI; icarcifriar20@gmail.com) under the voucher number CIFRI-MC22. The fins-clipped sample from fresh wild-caught *M. cavasius* fish was used for the present study. Later, following the manufacturer’s instructions, complete genomic DNA was isolated from the sample using the QIAGEN DNeasy blood and tissue genomic DNA isolation kit (Hilden, Germany) (cat. no. 69504). A spectrophotometer from NanoDrop was used to measure the amount of DNA, and gel electrophoresis evaluated its quality.

### Illumina library preparation, mitogenome sequencing, assembly, and annotation

The paired-end sequencing library was prepared using NEBNext^®^ Ultra™ DNA Library Prep Kit for Illumina^®^ NEB #E7645 (San Diego, CA). The library was put onto an Illumina NovaSeq 6000 for cluster formation and sequencing. FastQC was used to evaluate the raw reads and produce the quality read for further processing. *De novo* assembly of *M. cavasius* mitogenome was done using NOVOPlasty v4.2, CLC genomics workbench-6, and genome annotation using MITOS web server. A mitochondrial genome map was drawn using the GenomeVx webserver. The sequencing depth and coverage map of *M. cavasius* are provided in Supplementary Information Figure S1. Furthermore, phylogenetic analysis was performed by MEGA11.0 software (Tamura et al. 2021) and phylogenetic tree was better visualized using iTOL software (https://itol.embl.de/).

## Results and discussion

**Figure 2.**
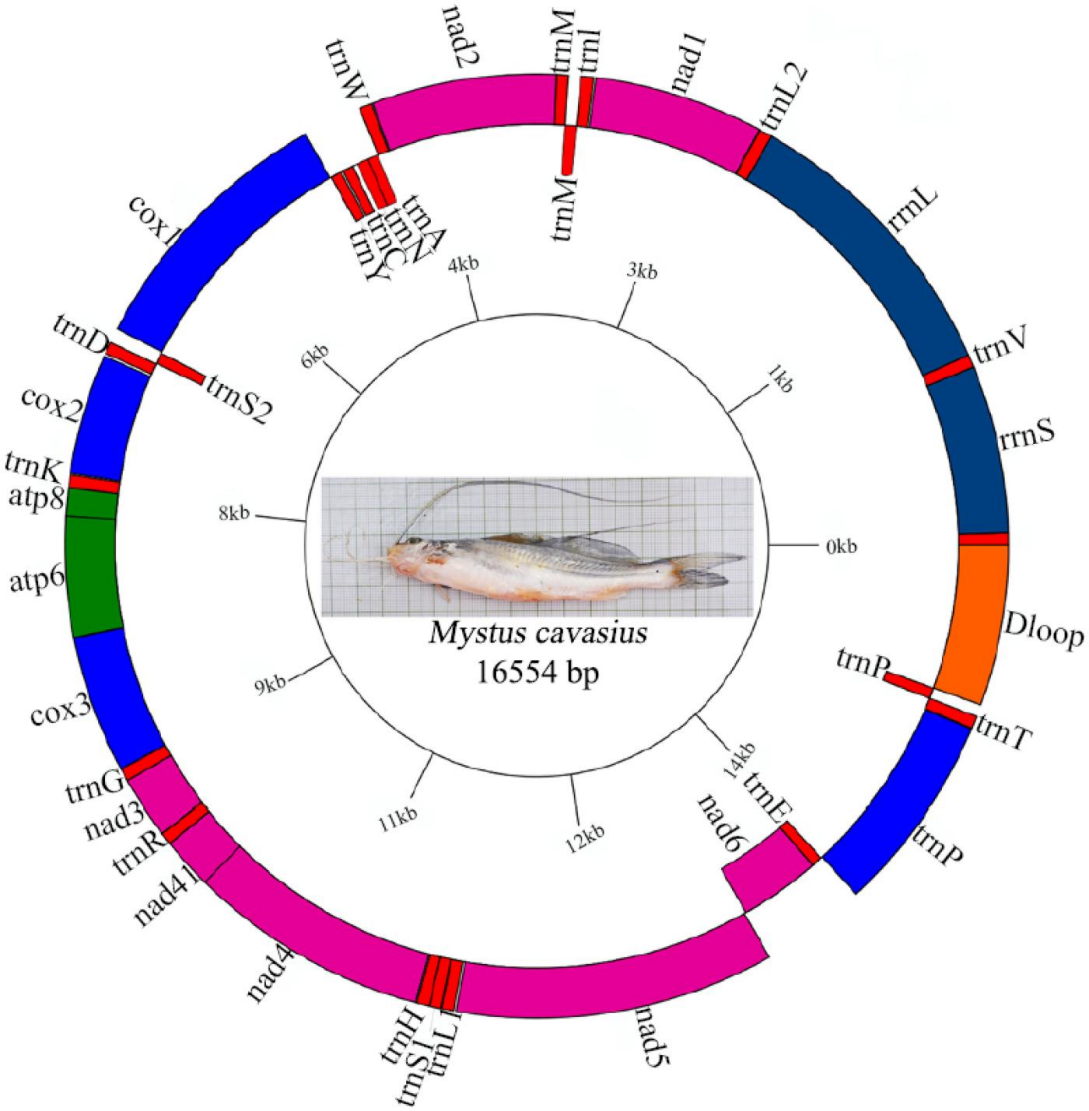
The illustrated map of complete mitochondrial genome of *M. cavasius.* A total of 37 genes including protein-coding genes (PCGs), rRNAs, tRNAs, and D-loop control region representing their location and features are displayed clockwise. Outer circle of circular map represents genes in heavy/positive strand and inner part represents genes in light/negative strand.

**Figure 3.**
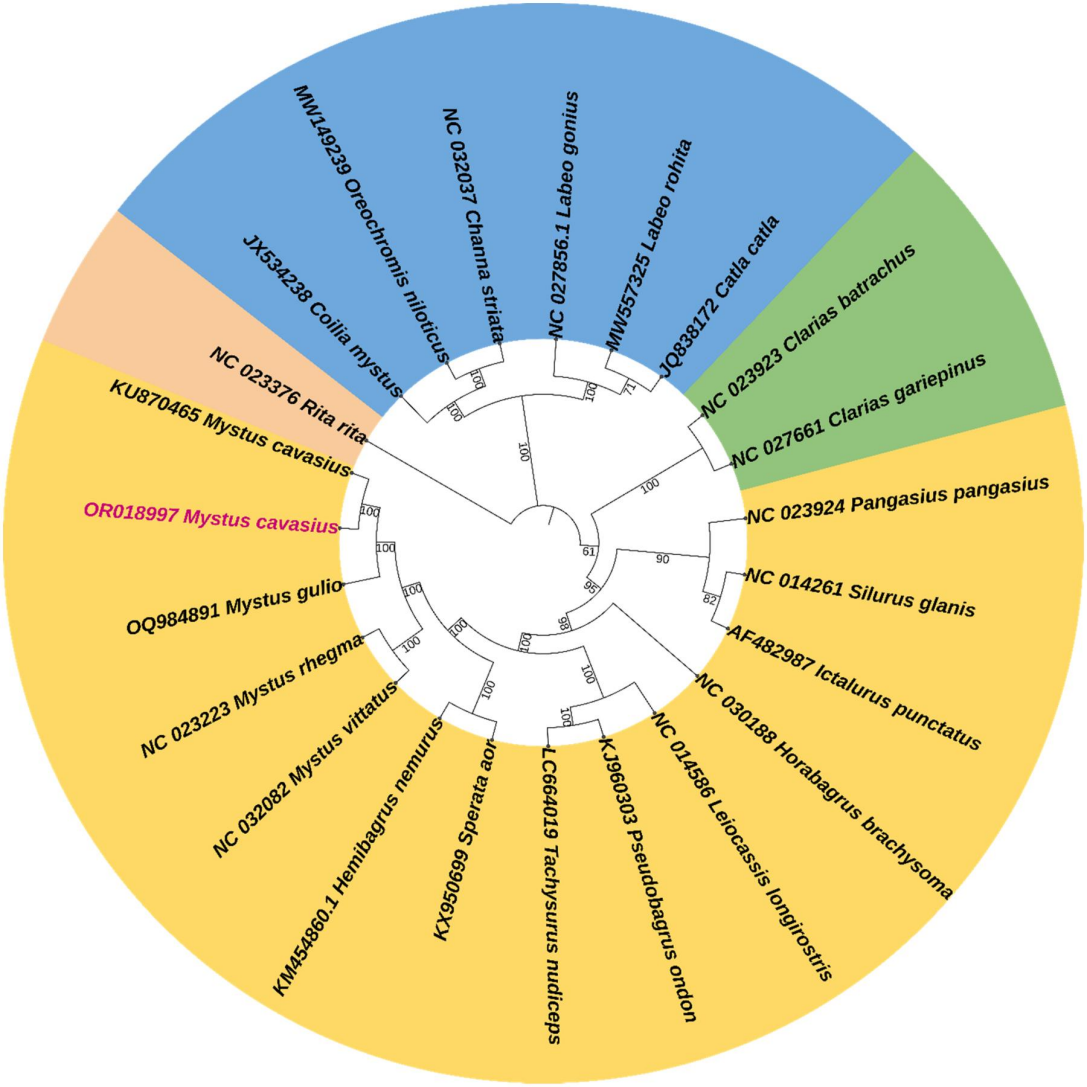
Maximum-likelihood phylogenetic tree and evolutionary analysis of *M. cavasius* using whole mitogenome sequence. The evolutionary distances were computed using the p-distance method and numbers at the nodes are bootstrap percent probability values based on 1000 replications. The phylogenetic tree was better visualized using iTOL software (https://itol.embl.de/). The following mitogenome sequences of fish were used: *M. cavasius* OR018997 (this study), OQ984891 (Roy et al. unpublished), KU870465 (unpublished), NC_023223, NC_032082, KM454860 (Wu et al. 2016), KX950699 (Lashari et al. 2016), LC664019, KJ960303, NC_014586 (Wang et al. 2011), NC_030188, AF482987 (Waldbieser et al. 2003), NC_014261 (Vittas et al. 2011), NC_023924, NC_027661 (Han et al. 2015), NC_023923, NC_023376, NC_032037, MW149239 (Nedoluzhko et al. 2021), JX534238 (Bo et al. 2013), and JQ838172 (Bej et al. 2012), MW557325.

Mitochondrial DNA has been extensively used as a marker for evolutionary, population genetic studies, phylogenetic inference, and global genetic barcoding initiatives due to their uniparental inheritance, lack of introns, high copy numbers, relatively simple structure, conserved gene composition, and rapid evolutionary rate (Ferreira and Rodriguez 2024; Roy et al. 2024). Over the past two to three decades, mitochondrial genes and genomes have been frequently used in fish and fisheries studies (Zhang et al. 2022). Mitochondrial sequence data have dramatically improved management and conservation; hence, the usefulness of the mitochondrial genome has received much attention in fisheries science (Lin et al. 2006). Complete mitochondrial genomes allow for greater flexibility in primer design and taxa-specific molecular assays that address eco-evolutionary questions relevant to management strategies (Ramón‐Laca et al. 2023; Alvarenga et al. 2024). In the present study, we have successfully sequenced and annotated the entire mitochondrial genome of *M. cavasius*. The complete *M. cavasius* mitochondrial genome comprised of 16,554 bp (GenBank accession number OR018997), which is well within the range of an average vertebrate mitogenome size of 15–20 Kb (Wang et al. 2019). The *M. cavasius* mitochondrial genome consists of a total of 37 genes, including 22 tRNA genes varing from 67 to 75 bp in length that code for 20 amino acids, two rRNAs (12S rRNA and 16S rRNA), 13 protein-coding genes (PCGs), which account for 68.50% of the entire mitochondrial genome, and a putative D-loop control region of 910 bp in length **(**Figure 2; Table 1 and Table S1). Most genes (28 genes) were encoded on the heavy (H) or ‘+’ strand. In contrast, nine genes, including eight tRNAs and a NADH dehydrogenase subunit 6 (ND6), were expressed on the other complementary light (L) or ‘–’ strand. Overall mitogenome nucleotide composition appears to be 31.94% adenine (A), 25.70% thymine (T), 14.93% guanine (G), and 27.43% cytosine (C), with A + T content (57.6%) being slightly higher than G + C content (42.4%) (Table S2). The complete structure of the mitogenome presented here could be a foundation for further research into population genetics, aquaculture genomics, phylogenetic analysis, and species identification of *Mystus* catfish and related species (Muhala et al. 2024). The phylogenetic relationship of *M. cavasius* was established based on the whole mitogenome sequences with closely related 16 bagrid catfishes and outgroups representing carps, clupeids, snakeheads, and cichlid fish species using the maximum-likelihood (ML) method. Results revealed that *M. cavasius* showed the closest relationship with all other *Mystus* species, including *M. gulio*, *M. cavasius*, *M. rhegma*, and *M. vittatus.* In addition, it clustered with other catfishes of family Bagridae, i.e. *Hemibagrus nemurus* and *Sperata aor*, with the highest bootstrap support value, i.e. 100% (Figure 3). Nevertheless, bagrid catfish *R. Rita* does not cluster with other bagrid species. The present finding was consistent with the previous report on the mitogenome phylogeny of *Mystus* and bagrid catfishes (Zhang et al. 2022; Nguyen et al. 2023; Chowdhury et al. 2024). This indicates the promising application of mitochondrial genome data in species taxonomy and phylogeny of bagrid fishes.

**Table 1.** List of annotated mitochondrial genes of *M. cavasius* and characteristic features.

Gene/element	From	To	Strand	Nucleotide (bp)	Anticodon
tRNA-Phe	1	70	+	70	TTC
12S ribosomal RNA	71	1022	+	952	
tRNA-Val	1023	1094	+	72	GTC
16S ribosomal RNA	1095	2769	+	1675	
tRNA-Leu	2769	2843	+	75	TTA
NADH dehydrogenase subunit 1	2847	3812	+	966	
tRNA-Ile	3823	3894	+	72	ATC
tRNA-Gln	3894	3964	–	71	CAA
tRNA-Met	3964	4033	+	70	ATG
NADH dehydrogenase subunit 2	4034	5071	+	1038	
tRNA-Trp	5079	5150	+	72	TGA
tRNA-Ala	5153	5221	–	69	GCA
tRNA-Asn	5223	5295	–	73	AAC
tRNA-Cys	5327	5393	–	67	TGC
tRNA-Tyr	5406	5476	–	71	TAC
Cytochrome c-oxidase subunit 1	5484	7019	+	1536	
tRNA-Ser	7029	7099	–	71	TCA
tRNA-Asp	7104	7173	+	70	GAC
Cytochrome c-oxidase subunit 2	7189	7872	+	684	
tRNA-Lys	7880	7953	+	74	AAA
ATPase subunit 8	7955	8119	+	165	
ATPase subunit	8113	8793	+	681	
Cytochrome c-oxidase subunit 3	8796	9578	+	783	
tRNA-Gly	9580	9652	+	73	GGA
NADH dehydrogenase subunit 3	9653	10,000	+	348	
tRNA-Arg	10,002	10,071	+	70	CGA
NADH dehydrogenase subunit 4 L	10,072	10,365	+	294	
NADH dehydrogenase subunit 4 L	10,362	11,735	+	1374	
tRNA-His	11,743	11,812	+	70	CAC
tRNA-Ser	11,813	11,879	+	67	AGC
tRNA-Leu	11,884	11,956	+	73	CTA
NADH dehydrogenase subunit 5	11,969	13,771	+	1803	
NADH dehydrogenase subunit 6	13,783	14,295	–	513	
tRNA-Glu	14,296	14,364	–	69	GAA
Apocytochrome b	14,373	15,500	+	1128	
tRNA-Thr	15,505	15,576	+	72	ACA
tRNA-Pro	15,575	15,644	–	70	CCA
Probable D-loop region	15,645	16,554		910	

## Conclusions

In conclusion, the study provides complete mitogenome data information, including gene content, structure, order, and phylogeny of *M. cavasius*. This work will contribute to the ongoing expansion of species representation in fish mitochondrial genome databases, facilitate the transition from single-gene metabarcoding approaches to multi-gene metagenomics methods for fish biodiversity assessment, and offer a helpful resource for phylogenetics, molecular ecology, conservation, and eDNA applications (Farrington et al. 2015; Schroeter et al. 2020).

## Supplementary Material

Supplementary.docx

## Data Availability

The mitogenome sequence data of *Mystus cavasius* in this study are openly available in the NCBI database (https://www.ncbi.nlm.nih.gov) under the GenBank Accession Number OR018997. The associated BioProject, Bio-Sample, and SRA numbers are PRJNA980942, SAMN35654811, and SRX20612709, respectively.
