# Supplementary material for: Whole mitochondrial genome sequencing and phylogenetic analysis of Gangetic mystus (Mystus cavasius)
Source: Mitochondrial DNA B Resour. 2024 Nov 15;9(11):1539–43. doi: 10.1080/23802359.2024.2427106 (PMC11571728; doi:10.1080/23802359.2024.2427106)
Supplement: Supplementary.docx [file TMDN_A_2427106_SM2396.docx]

**Supplementary files**

**
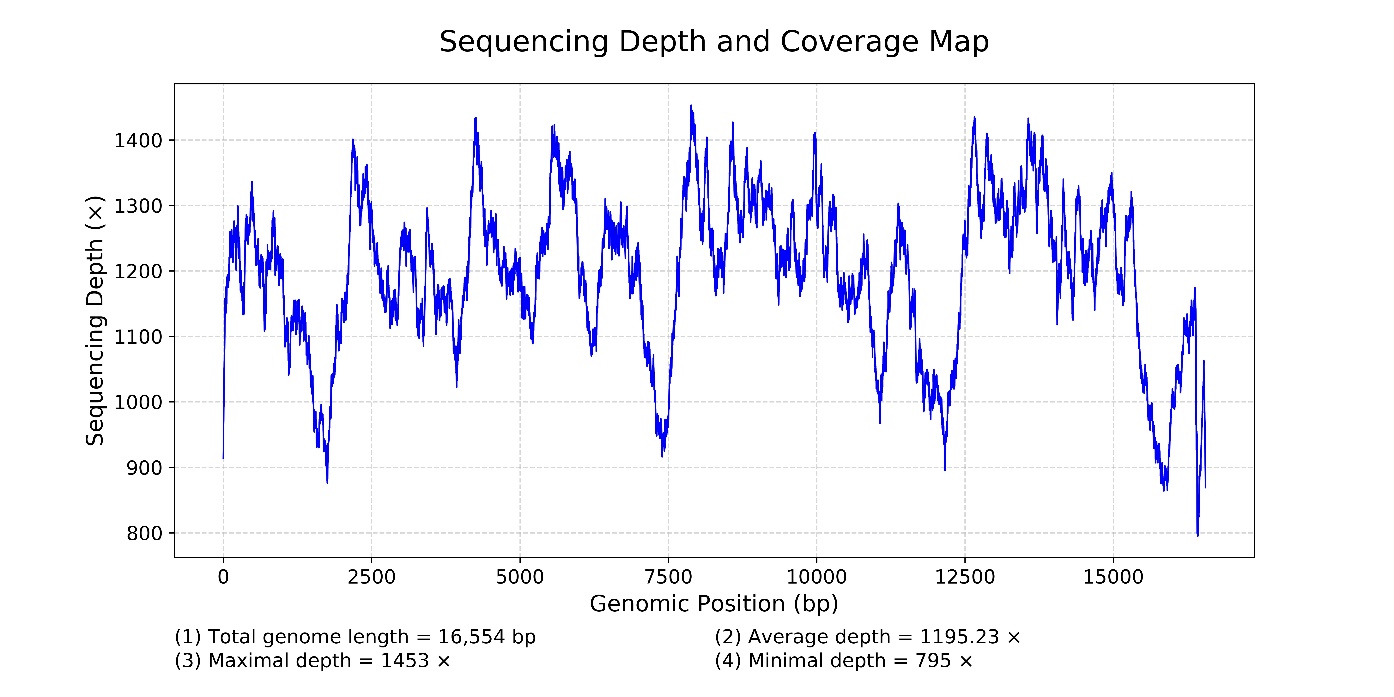
**

**Figure S1:** The sequencing depth and coverage map of *Mystus cavasius* (The map was generated according to Ni et al., 2023).

**Table S1: Nucleotide composition of sample *M. cavasius* mitochondrial genome**

| **PCGs** | **Start** | **Stop** | **Strand** | **Size of Nucleotide (bp)** | **Size of Amino Acid (aa)** |
| --- | --- | --- | --- | --- | --- |
| nad1 | 2847 | 3812 | + | 966 | 322 |
| nad2 | 4034 | 5071 | + | 1038 | 346 |
| cox1 | 5484 | 7019 | + | 1536 | 512 |
| cox2 | 7189 | 7872 | + | 684 | 228 |
| atp8 | 7955 | 8119 | + | 165 | 55 |
| atp6 | 8113 | 8793 | + | 681 | 227 |
| cox3 | 8796 | 9578 | + | 783 | 261 |
| nad3 | 9653 | 10000 | + | 348 | 116 |
| nad4l | 10072 | 10365 | + | 294 | 98 |
| nad4 | 10362 | 11735 | + | 1374 | 458 |
| nad5 | 11969 | 13771 | + | 1803 | 601 |
| nad6 | 13783 | 14295 | - | 513 | 171 |
| cob | 14373 | 15500 | + | 1128 | 376 |

**Table S2: Protein-coding genes and its characteristic features of *M.cavasius* mitogenome**

| **Content** | **bp** | **%** |
| --- | --- | --- |
| A% | 5287 | 31.94 |
| T% | 4255 | 25.7 |
| G% | 2472 | 14.93 |
| C% | 4540 | 27.43 |
| A+T% | 9542 | 57.6 |
| G+C% | 7012 | 42.4 |
| AT Skew | 0.1081 | |
| GC Skew | -0.2949 | |
